# Clinical image quality perception and its relation to NECR measurements in PET

**DOI:** 10.1186/s40658-014-0103-y

**Published:** 2014-12-23

**Authors:** Marcelo A Queiroz, Scott D Wollenweber, Gustav von Schulthess, Gaspar Delso, Patrick Veit-Haibach

**Affiliations:** Department of Medical Imaging, Division of Nuclear Medicine, University Hospital Zürich, Zürich, CH-8091 Switzerland; Department of Medical Imaging, Institute of Diagnostic and Interventional Radiology, University Hospital Zurich, Zurich, CH-8091 Switzerland; GE Healthcare, Waukesha, 53186 WI USA

**Keywords:** NECR, Image quality, FDG, Weight, BMI

## Abstract

**Background:**

The purpose of this study is to describe a clinical relation of noise equivalent count rate (NECR) - an objective measurement of positron emission tomography (PET) systems - measured in a large number of patients, to clinical image quality of PET and their relation to 18F-fluoro-2-deoxyglucose (FDG) activity and patient's weight.

**Methods:**

A total of 71 consecutive patients were evaluated in this retrospective study. All data was automatically analysed using Matlab to estimate the noise equivalent count rate. Then, image quality was evaluated according to two subjective scores: the *IQ local score* was a 3-point scale assigned to each bed position in all patients and the *IQ global score* was a 10-point scale assigned after evaluating the coronal whole-body PET. Patient data was also analysed concerning weight, body mass index, FDG dose at the start of acquisition (*D*_Acq_), presence of bowel uptake and presence of FDG-positive pathologic lesions. Two additional parameters were defined for each patient: the ratio between *D*_Acq_ and patient weight (*R*_DW_) and the ratio between *D*_Acq_ and patient BMI (*R*_DBMI_).

**Results:**

Clinically perceived image quality in PET has a significant positive correlation with NECR measured in patients, *R*_DW_, *R*_DBMI_ and presence of pathologic lesions. Clinical image quality furthermore has significant negative correlation with weight, body mass index (BMI) and presence of bowel uptake. Thresholds of *R*_DW_ and *R*_DBMI_ in which clinical IQ is good to excellent in more than 90% of the patients were 2.6 and 8.0, respectively.

**Conclusions:**

Clinically perceived image quality in PET systems is positively and significantly related to NECR measured in patients. An optimal threshold for the *R*_DW_ and *R*_DBMI_ was defined in which clinical IQ is good to excellent in more than 90% of patients. With this data, it is possible to extrapolate technical as well as clinical image quality to other PET system and to predict clinical image perception.

## Background

18F-fluoro-2-deoxyglucose (FDG) positron emission tomography (PET) has been worldwide established in oncologic imaging [1,2]. For this purpose, an accurate diagnosis is subject to the quality of the image, which can be influenced by FDG activity, uptake time, used PET reconstruction parameters (e.g. 2D vs. 3D, Time-of-Flight (TOF) vs. non-TOF) as well as acquisition time, patient's weight and blood sugar levels [3].

Assessing PET image quality (IQ) remains a challenge due to its clinical subjectivity and difficulties in standardisation. Over the years, there have been described at least four different methods to evaluate the PET image quality: image noise, noise equivalent count rate (NECR), NEMA NU-2 image quality phantom measurements and visual IQ.

The first two are automatic and objective measurements determined from the reconstructed image or PET raw emission data of phantoms.

Visual IQ assessed by imaging specialists represents a subjective evaluation of image contrast between different structures with different levels of noise [4]. There is only very limited literature available evaluating the relation between the NECR and visual IQ assessed by imaging specialists in the clinical routine with pathological findings in patients.

Ideally, the NECR applied here - measured directly in patients rather than in phantom experiments - could be used as a reliable tool for IQ quantification procedures in clinical protocols.

One study has compared the noise equivalent count density to visual IQ, showing that both are positively related. However, they used healthy volunteers, and thus, the clinical application might be limited [3].

Another study has evaluated the impact of patient's body mass index (BMI) on image quality. It has demonstrated that obese patients result in lower NECR and higher image noise which ultimately will lead to poorer image quality [5].

Recently, McDermott and co-authors have shown that noise equivalent count per axial length measured in the patient, noise equivalent count density and liver signal to noise ratio have a high discrimination for qualitatively assessed PET image quality [6].

Our study has been elaborated to describe the relation of NECR - an objective measurement of PET systems - measured in a large number of patients, to visual image quality of PET and their relation to FDG activity and patient's weight.

## Methods

### Patient population

A total of 71 consecutive patients (75 exams) were evaluated in this retrospective study. All patients were referred for a clinical FDG-PET/CT from January to December 2012 and underwent a PET/CT-MRI using a tri-modality setup. Based on the retrospective nature of the study, no formal institutional ethics committee approval is needed and the IRB was waived.

Exclusion criteria were uncontrolled glucose levels (higher than 120 mg/dL) and patients who have not fasted for at least 4 h, unwillingness to undergo the additional MR examination, claustrophobia and MR-incompatible medical devices (e.g. cardiac pacemaker, insulin pump, neurostimulator, cochlear implant). Another exclusion criterion was the presence of artefacts in at least one bed position on PET images (six exams in four patients were excluded), which made the clinical reading not applicable and could represent a confounding factor on IQ assessment.

Overall, 75 exams were performed in 71 patients (overall 655 bed positions; 41 males, 30 females; median age of 57 years, range 16 to 89 years). Of those, 59.2% (*n* = 42) presented with pathologic lesions (primary tumour, lymph nodes, metastasis and/or loco-regional recurrence), while 40.8% (*n* = 29) did not have any pathologic lesion. The indications for PET/CT were tumour primary staging in 22.5% (*n* = 16), follow-up or re-staging in 74.6% (*n* = 53) and 2.8% (*n* = 2) for non-oncologic reasons (fever of unknown origin and aortic aneurysm graft evaluation). Thirteen (18.3%) patients were under treatment during the exam.

### PET/CT imaging

PET/CT imaging was performed on a PET/CT-MR setup (including a time-of-flight Discovery 690 PET/CT and a Discovery 750w 3T MR; both from GE Healthcare, Waukesha, WI, USA).

PET/CT was performed according to the European Association of Nuclear Medicine (EANM) procedure guidelines for tumour PET imaging. Patients fasted for at least 4 h prior to injection of 18F-fluorodeoxyglucose (FDG). The mean FDG injected dose was 311.7 MBq (SD = 21.3, range 231.1 to 373.4). The mean FDG injected dose/body weight was 4.3 MBq/kg (SD = 0.9, range 2.6 to 6.6). Based on the additional MRI examination prior to the PET/CT, the mean delay between injection and PET scan was 95.7 min (SD = 12.7, range 69.9 to 128.3). Unenhanced low-dose CT and PET emission data were acquired from the mid-thigh to the vertex of the skull. CT data were acquired in shallow breathing with dose modulation between 15 and 80 mA, 120 kVp and a pitch of 0.984:1, reconstructed to images of 0.98-mm transverse pixel size and 3.75-mm slice thickness. PET data was acquired in 3D time-of-flight mode with a scan duration of 2 min per bed position, an axial FOV of 153 mm and a 23% overlap of bed positions, resulting in a total PET acquisition time ranging from 16 to 20 min. PET images were formed using a fully 3D TOF OS-EM iterative reconstruction including point-spread-function compensation (VuePoint-FXS) with 3 iterations and 18 subsets onto a 256 × 256 image grid (2.73 × 2.73 × 3.27 mm voxels) over a 700-mm-diameter FOV. Images were filtered in image space using a 4.0-mm FWHM in-plane Gaussian filter followed by an axial filter with a 3-slice kernel using relative weights of 1:4:1. All quantitative corrections, including normalisation, deadtime, randoms, scatter and attenuation, were applied during image reconstruction [7].

### Image processing and analysis

The acquired PET and CT images were transmitted to a dedicated review workstation (Advantage Workstation, GE Healthcare), which enables the review of the PET and CT images side by side or in fused/overlay mode (PET/CT).

In a first step, all data were automatically analysed using Matlab (MathWorks, Natick, MA, USA) to estimate the NECR versus activity concentration in a similar manner to the National Electrical Manufacturers Association (NEMA) analysis and based on previous publications [8-12]. This measurement is essentially the same usually performed on phantom data, but the required data (the number of true, scattered and random counts) are estimations either provided by the scanner software or automatically extracted from the reconstructed image. To determine the NECR for a bed position of patient data, the following steps were used: the PET raw data files were used to measure prompts and to calculate randoms from singles and obtain the bed position duration. Then, the reconstructed PET images were used to obtain an estimate of the model-based scatter fraction. With those two items, the NECR was calculated for each bed position, and the patient NECR was an average of all whole-body bed positions. By using the PET images, voxels above a fixed activity concentration of 1.0 kBq/mL, the average activity concentration for all voxels in the bed position was calculated. Decay to each bed position based upon its start time relative to scan start was applied as well.

While the measurement of NECR is a quantitative parameter, more clinical and qualitative evaluations were performed in a second step. Here, all the PET/CT exams were read qualitatively by a board-certified nuclear medicine physician/radiologist and a radiologist with substantial experience in PET/CT image reading.

Two subjective scores were defined to evaluate image quality. The *IQ local score* (IQ_L_) was a 3-point scale assigned to each bed position in transversal planes in all patients (*n* = 655 bed positions), where 1 means poor, 2 means good and 3 means excellent IQ. The *IQ global score* (IQ_G_) was a 10-point scale assigned after evaluating the coronal whole-body PET, where 1 was the worst IQ and 10 was the best. The readers assessed the image quality subjectively based on prevalence of noise, contrast between different tissues and organs (e.g. differentiation between the lung and thoracic wall) and lesion detectability. There was no dedicated evaluation of each of those parameters independently (noise, contrast and detectability).

The advantage of performing both scores remains on the possibility to evaluate the whole-body at one time (IQ global score) and in a more detailed approach, identifying better the relation between the different anatomical structures within the body compartments (IQ local score).

Patient data were analysed concerning overall weight, BMI, FDG dose at the start of acquisition (*D*_Acq_), presence of bowel uptake and presence of FDG-positive pathologic lesions (significantly higher uptake than the surrounding background activity).

For the qualitative evaluation of focal bowel uptake and pathologic FDG-positive lesions, only the bed stations, which showed those lesions, were analysed (e.g. only the two abdominal bed stations were analysed concerning focal bowel uptake and were correlated with the IQ local score).

Two additional parameters were defined for each patient: the ratio between the dose at the start of acquisition (*D*_Acq_) and patient weight (*R*_DW_) and the ratio between *D*_Acq_ and patient BMI (*R*_DBMI_). The *R*_DW_ and *R*_DBMI_ thresholds were determined, above which the resulting image quality was at least good (IQ_L_ > 2) in more than 90% of patients. The rationale behind these parameters was to create a threshold for different weight/BMI groups with which we could determine the dose needed to achieve high IQ scores throughout our patient group.

Our population was divided into four different groups according to their BMI: group I (BMI lower than 20), group II (BMI between 20.1 and 25), group III (BMI between 25.1 and 30) and group IV (BMI higher than 30.1). BMI groups were analysed concerning the differences in NECR and IQ_L_.

### Statistical analysis

All statistical tests were performed using SPSS Statistics Version 21 (IBM, Armonk, NY, USA). *p* values <0.05 were considered statistically significant. Pearson's correlation analysis was performed to evaluate the relation between NECR, IQ_L_, IQ_G_, weight, BMI, *R*_DW_ and *R*_DBMI_. One-way ANOVA test was performed to calculate the difference between the four different BMI groups. Tukey's test was additionally applied to evaluate the differences between each BMI group. Mann-Whitney test was applied to analyse the difference of NECR and IQ_L_ in patients with bowel uptake and pathologic lesions.

## Results

The main histologic types of tumours were lymphoma and lung and breast cancer. Patients and tumour characteristics are summarised in Table 1.

**Table 1:** **Patient and tumour characteristics**

	**Value**
Number of patients	71
Pathologic lesion, *n* (%)	
Yes	42 (59.2)
No	29 (40.8)
Indication, *n* (%)	
Primary staging	16 (22.5)
Follow-up/re-staging	53 (74.6)
Non-oncological (FUO and aortic aneurysm graft)	2 (2.8)
Tumour type, *n*	
Lymphoma (NHL/HD)	18 (11/7)
Lung cancer (proved/suspicious/incidental)	13 (10/2/1)
Breast cancer	9
Melanoma	7
Renal cell	3
Oesophagus, larynx, stomach	2
Adrenal, anal, colon, fibrosarcoma, follicular thyroid, hypopharynx, nasopharynx, oropharynx, ovarian, paraganglioma, myeloid sarcoma, mesothelioma, rectal, SCC right upper arm	1
Under therapy, *n* (%)	
Yes	13 (18.3)
No	56 (78.9)
N/A	2 (2.8)

FUO, fever of unknown origin; NHL, non-Hodgkin lymphoma; HL, Hodgkin lymphoma; SCC, squamous-cell carcinoma; N/A, not applicable.

Descriptive mean data for the IQ_L_, IQ_G_, NECR, weight, BMI, injected dose, injected dose/body weight and ranges of all values are summarised in Table 2.

**Table 2:** **Overall PET and patient parameters and per BMI group (group I < 20, group II = 20.1 to 25, group III = 25.1 to 30 and group IV > 3)**

**Parameter**	**Overall**			**Group I**			**Group II**			**Group III**			**Group IV**		
	**Mean**	**Min**	**Max**	**Mean**	**Min**	**Max**	**Mean**	**Min**	**Max**	**Mean**	**Min**	**Max**	**Mean**	**Min**	**Max**
IQ_L_	2.1	1.44	2.88	2.47	2.13	2.88	2.23	1.89	2.78	1.99	1.6	2.44	1.82	1.44	2.44
IQ_G_	7.3	5	9.5	8.38	7.5	9.5	7.72	7	9	7	6	8	6.38	5	8
NECR (kcps)	106.4	42.2	323.5	133.04	116.5	150.7	112.49	85	140.7	102.34	63.6	129.7	86.79	67.4	11.7
Weight (kg)	75.3	45	126	52	45	57	67.91	50	87	80.52	69	100	95.62	72	126
BMI (kg/m^2^)	25.9	16.1	43.6	18.6	16.14	19.96	22.87	20.2	24.77	27.28	25.16	29.73	34.08	30.47	43.6
Injected dose (MBq)	311.7	231.1	373.4	307.13	231.07	322.6	316.09	237.9	368.8	310.6	275.01	373.45	315.82	296.8	343.7
Injected dose/body weight (MBq/kg)	4.3	2.6	6.6	5.94	5.13	6.61	4.73	3.55	6.25	3.9	3.1	4.76	3.37	2.58	4.14
Uptake time (min)	95.7	69.9	128.3	94.88	74.53	116.78	93.91	74.43	117.1	95.57	69.9	128.33	100.08	82.22	117.77

**Figure 1:**
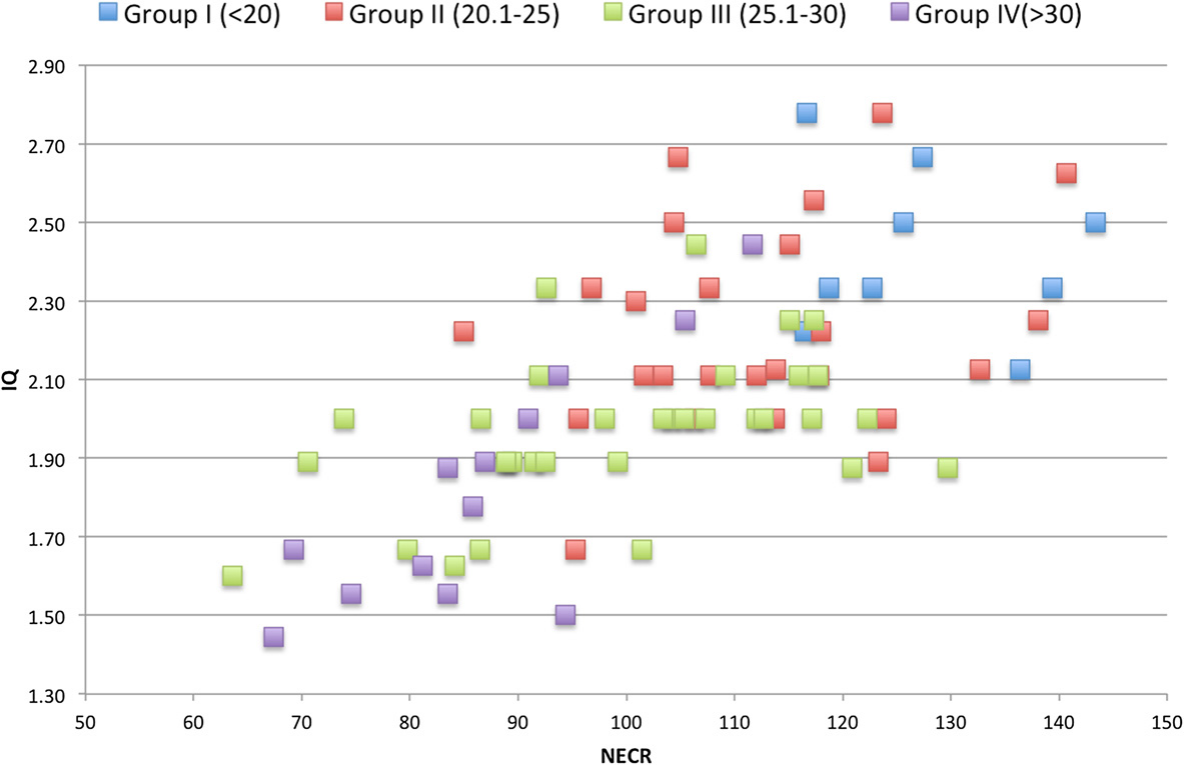
**Graphic showing the relation between IQ**_**L**_ **and NECR (kcps) per BMI groups.**

IQ_L_ showed moderate but statistically significant correlation with NECR (*r* = 0.64 and *r* = 0.67, respectively, *p* value <0.01) (see Figure 1). The mathematical functions for which correlate IQ_L_ and NECR for the entire data and for each BMI group (from I to IV) of patients are as follows: *y* = 0.0106*x* + 0.9734, *y* = 0.0049*x* + 1.8259, *y* = 0.0045*x* + 1.7194, *y* = 0.0056*x* + 1.4105 and *y* = 0.0197*x* + 0.1126.

**Figure 2:**
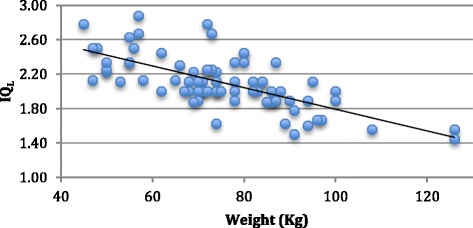
**Graphic showing the relation between IQ**_**L**_ **and weight.**

**Figure 3:**
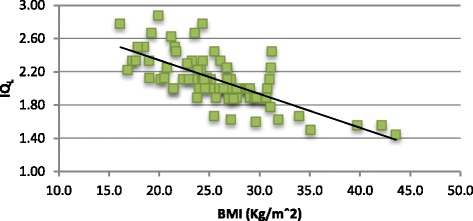
**Graphic showing the relation between IQ**_**L**_ **and BMI.**

There was a significant negative correlation between IQ_L_ and weight and BMI (*r* = 0.69 and *r* = 0.71, respectively, *p* value <0.01) and IQ_G_ and weight and BMI (*r* = 0.75 and *r* = 0.77, respectively, *p* value <0.01), represented in Figures 2 and 3.

**Figure 4:**
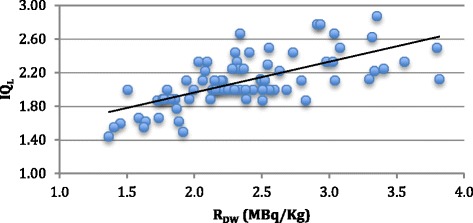
**Graphic showing the relation between IQ**_**L**_ **and** ***R***_**DW**_ **.**

**Figure 5:**
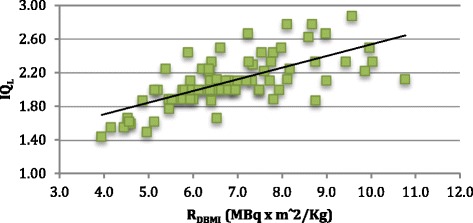
**Graphic showing the relation between IQ**_**L**_ **and** ***R***_**DBMI**_ **.**

**Figure 6:**
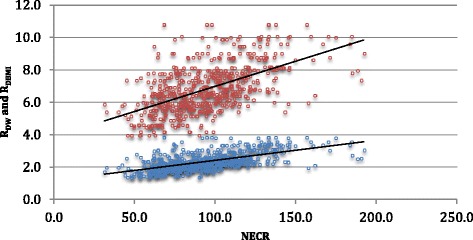
**Graphic showing the relations between** ***R***_**DW**_ **(blue circle) and** ***R***_**BMI**_ **(red circles) and NECR.**

The *R*_DW_ and *R*_DBMI_ were significantly correlated with IQ_L_ (*r* = 0.67 and *r* = 0.68, respectively, *p* value <0.01), IQ_G_ (*r* = 0.74 and *r* = 0.74, respectively, *p* value <0.01) and NECR (*r* = 0.89 and *r* = 0.84, respectively, *p* value <0.01). These findings are presented in Figures 4, 5 and 6.

Among the patients who presented bowel uptake, there was no significant difference between NECR (98.4 vs. 102.8, *p* = 0.318), but there was significant difference for IQ_L_ (1.85 vs. 2.01, *p* = 0.032).

On the other hand, patients who presented at least one pathologic lesion showed significantly higher IQ_L_ (2.16 vs. 1.94, *p* = 0.001) and NECR (105.0 vs. 92.6, *p* = 0.001).

The thresholds calculated for *R*_DW_ and *R*_DBMI_ were 2.6 and 8.0, respectively.

**Figure 7:**
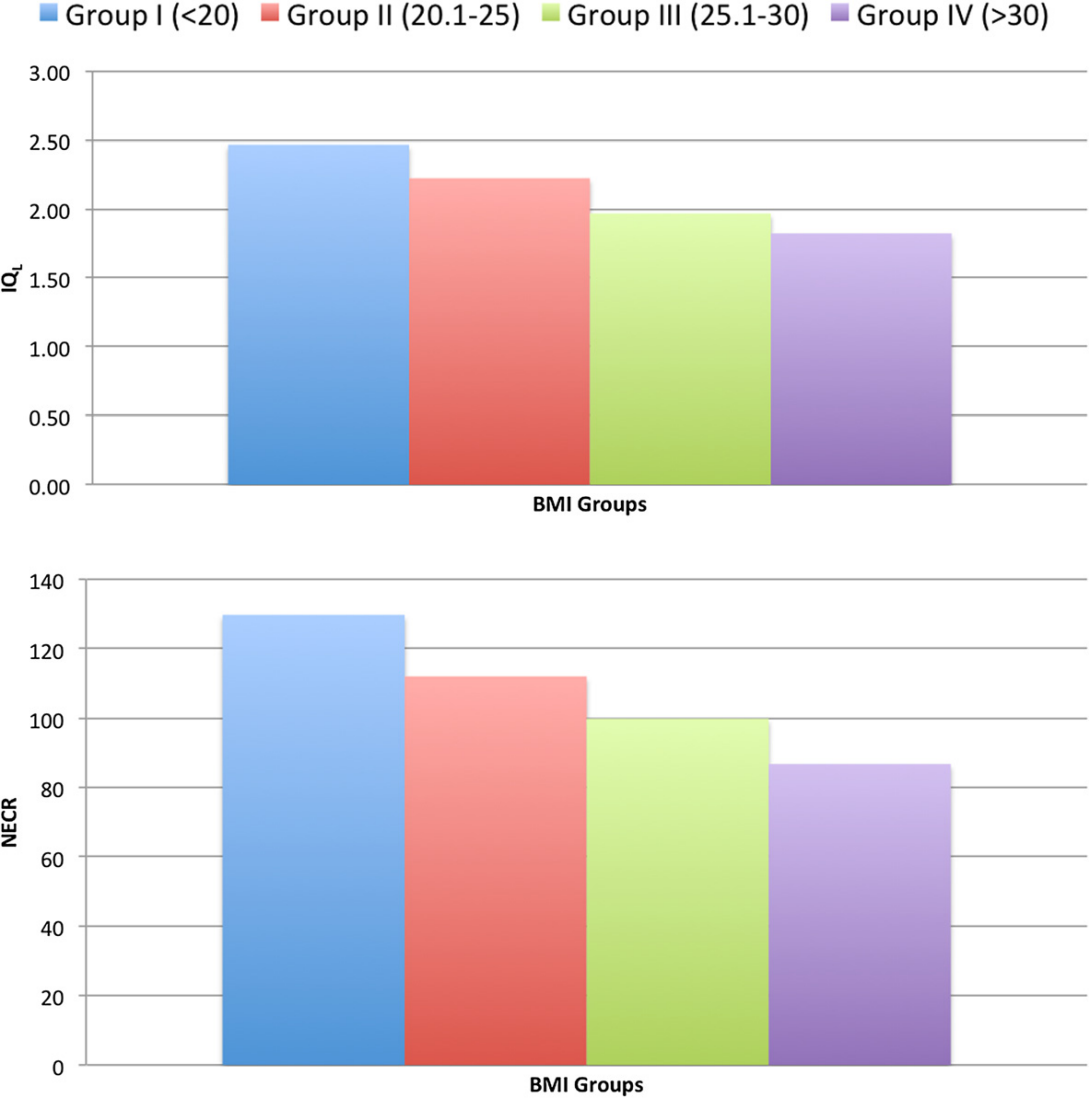
**Graphics showing the relations between IQ**_**L**_ **and NECR per BMI groups.**

Concerning NECR, there was a statistically significant difference between the four BMI groups (*p* < 0.01), except between groups II and III. Concerning IQ_L_, there was a statistically significant difference between the four BMI groups (*p* < 0.01), except between groups III and IV (see Table 3). A graphic showing the relations of IQL average and NECR average per BMI groups and the correlation of IQL and NECR for all BMI groups are represented in Figure 7.

**Table 3:** **NECR and IQ**_**L**_ **variation according to patient's BMI**

**BMI groups**	**I (<20)**	**II (20 to 25)**	**III (25 to 30)**	**IV (>30)**	***p*** **value**
NECR	129.75	112.49 ^a^	102.34 ^a^	86.79	<0.05
IQ_L_	2.47	2.23	1.99 ^b^	1.82 ^b^	<0.05

Different letters (a, b) mean not statistically significant from each other.

## Discussion

This is one of the first studies showing that clinically perceived image quality in PET has a significant positive correlation with NECR measured in patients, *R*_DW_, *R*_DBMI_ and presence of pathologic lesions. Clinical image quality furthermore has a significant negative correlation with weight, BMI and presence of bowel uptake. An optimal threshold for the *R*_DW_ and *R*_DBMI_ in which clinical IQ is at least good in more than 90% of the patients has been defined as well. Lastly, our conclusions (e.g. recommended FDG dose) are available both in terms of perceptual diagnostic image quality and of automated technical image quality measures. This means that our results can be rescaled to be applied to any other PET scanner.

### Image quality

In this study, the methods used for the evaluation of IQ were found to be reliable for two main reasons. First, because both the IQ_L_ and IQ_G_, although independent methods, were significantly and strongly correlated to each other (*r* = 0.94). The second reason was the significant and positive correlation between IQ_L_ and IQ_G_ to NECR, a well-established and quantitative way of assessing IQ of PET systems [3,5].

There were no images scored below 5 on IQ_G_ or below 1.44 on IQ_L_, because all the patients were referred to a clinical PET evaluation and, therefore, required an image with a diagnostic quality.

### NECR

NECR measurements represent a method of approximation to image quality in PET systems, at least to a certain extent which seems clinically reliable. However, getting the NECR values is a challenge. Several studies have used NECR based on phantom or simulation measurements [9,11,12]. One study has measured NECR in a patient population [5]; however, they compared this value only with image noise (a different automatic technical measurement). One major difference compared to our study is that our estimation of NECR is patient-based and that an additional correlation was made to visual IQ assessments. Thus, we actually have a clinical image estimation, which can be transferred to other PET/CT systems with different detector technologies to compare and estimate the expected clinical IQ. NECR measurements are already widely accepted and used as means of comparing data acquisition performance between different PET systems. By establishing a link between our clinical (subjective) evaluation and the corresponding raw data quality in terms of NECR, it is indeed possible to estimate the tracer doses required to achieve similar results in other scanners. This can be achieved by comparing the phantom-based NECR curves provided by the respective manufacturers.

### Correlation to clinical perception

Both IQ_L_ and IQ_G_, as well as NECR, showed a significant positive correlation with the *R*_DW_ and *R*_DBMI_.

These results are partially in accordance with the literature. Everaert et al. showed that administration of activities of FDG of ≥8 MBq/kg results in PET images of good to excellent quality in the vast majority of patients using a standardised protocol [4]. On the other hand, Chang and co-workers have demonstrated that an increase in injected dose means a (scanner-dependent) raise in NECR, but only until it reaches the known plateau. From this point on, there is no difference on NECR by increasing FDG injected dose anymore [5].

Our study showed an optimal threshold of 2.6 MBq/kg (*R*_DW_) in which the images are equal or above good IQ. The threshold we found is lower than that published in the literature, because we used the FDG dose during the acquisition of PET (after the uptake time). That holds the advantage that it is not influenced by the delay between injection and PET exam. Using a standardised uptake time of 60 min, the *calculated* threshold in our setup value would be 3.8 MBq/kg. However, this is still lower than that published in other studies [9]. Since IQ, NECR and *R*_DW_ are well correlated with each other, the threshold of *R*_DW_ below the clinical image quality is suboptimal and can be translated into a corresponding NECR threshold, which then identifies such suboptimal image quality in a quantitative way.

The EANM recommends standardised FDG activity. For systems with a high count rate capability, the administered FDG activity and scan duration for each bed position should be a relation between the injected dose/kg and time per bed position. Therefore, one may decide to apply a higher activity and reduce the duration of the scan or use reduced activity and increase scan duration - depending on the clinical situation [13].

Putting our technical parameters and clinical evaluations in accordance with those recommendations, the FDG activity would be 6.9 MBq/kg. Our study has shown that PET imaging quality remains good to excellent in the majority of patients even using significantly lower dose.

### Weight and BMI

Our study has demonstrated that weight and BMI are negatively and significantly correlated to IQ and NECR. This is expected since other studies have already established this relation. By increasing the patient's weight and BMI, the image quality deteriorates and NECR decreases significantly. This is explainable by the standardised fixed dose of 350 MBq (±10%) that was used in our department at the time period when this study was performed.

As described before, we have also found that in patients with higher BMI, higher FDG doses are needed in order to increase NECR and, hence, image quality. However, it has been shown that once the NECR curve is approaching its plateau it is not useful to increase the injected dose [5]. Furthermore, there are maximal FDG activities defined by the national laws that can be applied [13]. Thus, in current clinical systems (e.g. lutetium oxyorthosilicate (LSO)), FDG activities higher than 530 MBq should not be administered [14]. To increase the image quality in patients where the maximal FDG dose is already given, it is recommended to increase the time per bed position instead of giving higher doses [13].

Our results concerning perceptual and technical differences between weight/BMI groups are also in accordance with the current literature. Recently, Chang and co-workers have demonstrated statistical differences in NECR and signal-to-noise ratio between two different BMI groups [15]. In our population, there was no statistical difference between the BMI groups II and III regarding NECR, while concerning IQ_L_, there was no statistical difference between the groups III and IV. One possible explanation for this might be the different appreciation of the image quality between automatic and subjective criteria. In this case, it would appear that the subjective perception of image quality loss with larger patients tends to ‘saturate’ as BMI increases.

### Presence of FDG bowel uptake

In clinical routine, partly very high FDG uptake can be seen in the gastrointestinal tract, e.g. in the distal oesophagus, stomach, small intestine and large intestine representing normal patterns of tracer distribution (due to diabetes medication) or inflammatory disease [16]. Diffuse increased FDG uptake in these areas is defined as physiologic and unrelated to the malignant process with relatively high certainty [17]. However, especially medication-related high bowel uptake in patients with diabetes can sometimes impair reading in PET/CT clinical routine.

Our study has found that the presence of bowel uptake is related with (significantly) lower IQ scores, but this relation could not be established for NECR measurements. Thus, although high bowel uptake subjectively impairs the clinical reading, those differences are not reflected within the quantitative measurements.

Our study has shown that the presence of focal FDG uptake, malignant or not, is related to high IQ scores and high NECRs. The reason for the first might be the subjective analysis as well. The presence of FDG focal uptake increases the soft tissue contrast between the structures, enhancing therefore the visual IQ.

### Limitations

Our study has several technical limitations. Our data shows a partial correlation between NECR and IQ on average, however, with partially high dispersion. This dispersion of IQ values for a given value of NECR is probably related to the subjective nature of the IQ itself. Although we already performed consensus reading, a given subjectivity cannot be excluded.

Relatively large variations in IQ occur for particular values of NECR. However, the intent of the manuscript was to show a clinically usable approximation between NECR and image quality. This is also why the transferability of our results is not of a quantitative but rather qualitative nature, which is thought to be clinically usable and useful.

## Conclusions

Clinically perceived image quality in PET is positively and significantly related to quantitative NECR measured in patients. An optimal threshold for the *R*_DW_ and *R*_DBMI_ was defined in which clinical IQ is good to excellent in more than 90% of patients. With this data, it is possible to extrapolate technical as well as clinical image quality to other PET system and to predict clinical image quality perception.
